# The relationship between caffeine consumption and colon cancer prevalence in a nationally representative population

**DOI:** 10.3389/fnut.2024.1375252

**Published:** 2024-05-28

**Authors:** Yuhua Qu, Yulu Cheng, Fengming Chen

**Affiliations:** ^1^Department of Anorectology, Hospital of Chengdu University of Traditional Chinese Medicine, Chengdu, China; ^2^Department of Disinfection Supply Center, Hospital of Chengdu University of Traditional Chinese Medicine, Chengdu, China

**Keywords:** caffeine intake, colon cancer, dose–response relationship, restricted cubic spline, NHANES

## Abstract

**Aims:**

This study examines the correlation between caffeine consumption and the prevalence of colon cancer.

**Methods:**

Utilizing data from the National Health and Nutrition Examination Survey (NHANES) for the years 2001 to 2014, we applied weighted logistic regression to evaluate the association between caffeine consumption and the prevalence of colon cancer. This analysis accounted for variables including age, gender, race, education, poverty income ratio, smoking status, alcohol consumption, and diabetes. The findings were expressed as weighted odds ratios (ORs) with accompanying 95% confidence intervals (CIs). The restricted cubic spline analysis was performed to exam the dose-dependent relationship.

**Results:**

The study included 27,637 participants, of which 144 were diagnosed with colon cancer and 27,493 served as controls. Individuals in the highest quartile (Q4) of caffeine consumption (Q4) displayed a significantly increased risk of colon cancer compared to those in the lowest quartile (Q1), with a weighted OR of 2.00 (95% CI: 1.11–3.59; *p* = 0.022). Additionally, restricted cubic spline analysis indicated a significant correlation between higher caffeine intake and increased colon cancer risk, with an overall association *p*-value of 0.007.

**Conclusion:**

These findings suggest a potential relationship between higher levels of caffeine consumption and an increased risk of colon cancer. The dose–response relationship suggests a notable correlation at higher caffeine intake levels. Further investigations are warranted to confirm these results and elucidate potential underlying mechanisms.

## 1 Introduction

Coffee is one of the most commonly consumed beverages worldwide, with an estimated yearly trade of more than 10 billion dollars (1). Its global popularity stems from its distinctive flavor and stimulating properties, making it a staple in the daily routines of billions (2). On average, an American adult consumes about 1.5 standard cups of coffee daily, making caffeine a major component of their daily intake (3). Coffee contains a variety of bioactive compounds linked to several health conditions, including diabetes (4), cardiovascular disease (5), cancer (6), and so on. These compounds include caffeine, chlorogenic acids, and various minerals, all of which can significantly impact human health (7). Among the many biological compounds, caffeine, chemically known as 1,3,7-trimethylxanthine, mirrors the molecular structure of adenosine, enabling it to block adenosine receptors and inhibit its effects (8). Notably, caffeine has been shown to exhibit antioxidant and anti-inflammatory properties, modulate the gut microbiome, and alter drug/nutrient metabolism (9). Therefore, the potential health impacts of caffeine consumption have become a focal point of extensive research.

Colon cancer, also referred to as colorectal cancer, is a malignancy originating in the colon or rectum. As the third most common cancer, colon cancer has become the second leading cause of cancer-related deaths worldwide, posing a significant public health concern (10). The causes of colon cancer are multifactorial, with lifestyle factors playing a significant role in its onset and progression (11). The impact of caffeine intake on the risk of digestive tract cancers has been extensively studied, yet the findings remain inconsistent. Some research indicates a protective effect of caffeine, suggesting it may lower the risk of certain digestive tract cancers (12, 13), while other studies have found no significant relationship (14–16). The disparity underscores the need for further research to clarify the role of caffeine consumption in the development of colon cancer.

Given the widespread consumption of coffee and caffeine among U.S. adults, this study investigates the potential association between caffeine intake and the prevalence of colon cancer in US population. We analyzed data from large-scale, population-based surveys, taking into account numerous potential confounding factors. Our findings aim to enrich the existing body of knowledge regarding the health effects of caffeine consumption and may have significant implications for public health recommendations and prevention strategies for colon cancer.

## 2 Methods

### 2.1 Study population

The National Health and Nutrition Examination Survey (NHANES) is a large, nationally representative, cross-sectional survey conducted by the Centers for Disease Control and Prevention in the United States (17). NHANES is designed to investigate the health and nutritional status of the non-institutionalized US population via interviews, questionnaires, physical examinations, and laboratory tests. The survey protocol, including study design and data collection procedures, is approved by the National Center for Health Statistics Ethics Review Board, and all the participants provided the written informed consent. The data were accessed for research purposes on 02/04/2023.

NHANES has provided nationally representative samples to explore the association between caffeine intake and colon cancer. Our study used data from seven consecutive NHANES two-year cycles, spanning from 2001 to 2014. These cycles were selected to ensure a sufficient sample size and to allow for the analysis of trends over time. The NHANES survey employs a complex, multistage probability sampling design, ensuring the representation of various demographic groups, including age, sex, race/ethnicity, and socioeconomic status. The inclusion criteria for our study population were adults aged ≥20 and < 80, and individuals with missing data on mobile examination center collected caffeine consumption or cancer status were excluded to reduce potential bias in the analysis. According to the inclusion and exclusion criteria, the final study population comprised a total of 27,637 participants. The NHANES sampling weights were utilized for the survey design to guarantee the nationally representative estimate for the US population.

### 2.2 The identification of colon cancer

To identify participants with a history of colon cancer, we relied on self-reported medical data from questionnaires. In the continuous NHANES questionnaire survey, participants answered the following two questions: ‘Have you ever been told by a doctor or health professional that you have colon cancer?’, and ‘Have you ever been told by a doctor or health professional that you have rectum cancer?’. A positive response to any of these questions indicated a self-reported history of colon cancer. To minimize the potential misclassification bias, we considered participants to have a history of colon cancer only when they reported a positive diagnosis for colon or rectum cancer.

### 2.3 Caffeine intake assessment

Caffeine intake in our study was assessed using the Dietary Interview – Total Nutrient Intakes data from the NHANES survey. The dietary intake information was obtained through in-person 24-h dietary recall interviews, conducted by trained interviewers based on the United States Department of Agriculture’s (USDA) Automated Multiple-Pass Method. This method is designed to ensure the accuracy of self-reported dietary intake by guiding respondents through a structured, detailed interview process. To estimate caffeine intake, the reported food and beverage consumption data were linked to the USDA Food and Nutrient Database for Dietary Studies (FNDDS). The FNDDS provides detailed information on the nutrient content of various food items, including caffeine levels. The daily caffeine intake for each participant was calculated by summing up the caffeine content of all consumed food and beverages during the 24-h recall period.

We utilized only the First Day Dietary Interview data to estimate caffeine intake. The First Day Dietary Interview was performed in a mobile examination center, where participants were interviewed in a controlled environment (18). This approach ensures consistency and accuracy in the data collection process. The decision to use only the first day of dietary recall was based on the assumption that it provides a reliable snapshot of the participants’ habitual caffeine consumption while minimizing the potential for recall bias.

### 2.4 Covariates

In this study, we adjusted for several potential confounding factors that could influence the association between caffeine intake and the prevalence of colon cancer. The covariates included in our analyses were age (continuous), gender (male or female), race/ethnicity (categories), education level (categories), poverty income ratio (categories), smoking status (never smokers, former smokers, and current smokers) (19), alcohol consumption (yes/no), and diabetes (yes/borderline/no). These covariates were incorporated into the statistical models to account for their potential effects on the relationship between caffeine intake and the prevalence of colon cancer. Education level is often used as a proxy for socioeconomic status and may be associated with cancer risk through various mechanisms, such as health behaviors and access to healthcare. We categorized education into three groups: low high school, high school, and above high school. Poverty income ratio is a measure of socioeconomic status based on the ratio of family income to the poverty threshold. It is a continuous variable that may be associated with cancer risk through factors such as access to healthcare, health behaviors, and environmental exposures. Poverty income ratio was categorized into three levels, including <1.33, 1.33 ~ 3.5, and ≥ 3.5. In this study, participants who consuming at least 12 alcohol drinks a year were defined as drinkers (20).

### 2.5 Statistical analysis

This study used the sample weight (WTDRD1/7) to account for the complex survey design considering stratification, clustering, and oversampling. The sample weight was divided by 7 to adjust for the use of seven consecutive two-year cycles from 2001 to 2014, ensuring that the results are representative of the US adult population. To compare the demographic, lifestyle, and dietary characteristics between cancer patients and controls, we conducted descriptive analyses. Continuous variables were presented as weighted means ± standard errors, while categorical variables were presented as weighted proportions. We performed weighted t-test for continuous variables and weighted chi-square test for categorical variables to determine the differences between the groups.

To investigate the association between caffeine intake and the prevalence of colon cancer, we utilized weighted logistic regression controlling for various potential confounders. Three distinct models were employed: Model 1 is the unadjusted model; Model 2 is adjusted for age, gender, and race; and Model 3 is the fully adjusted model, which includes adjustments for age, gender, race, education, poverty income ratio levels, smoking, drinking, and diabetes. The caffeine intake was first analyzed as a continuous variable. It was subsequently categorized into quartiles of intake for further analysis, with the groups defined as: ≤13 mg, 13 ~ 97 mg, 97 ~ 213 mg, and > 213 mg. The results were presented as weighted odds ratio (OR) with corresponding 95% confidence interval (CI). Additionally, we employed restricted cubic spline regression with four knots to further investigate the dose–response relationship between caffeine intake and colon cancer.

All statistical analyses were performed based on R software, and *p* < 0.05 was considered statistically significant.

## 3 Results

### 3.1 Participant characteristics by cancer status

27,637 participants were enrolled in the study, with 144 individuals in the colon cancer group and 27,493 individuals in the control group. The baseline characteristics by cancer status are shown in Table 1. The average age of the cancer group was significantly higher than that of the control group (64.67 ± 1.17 vs. 45.44 ± 0.24, *p* < 0.001). Caffeine intake was also significantly different between the two groups, with the cancer group having higher mean caffeine intake (241.51 ± 18.96 mg) compared to the control group (183.08 ± 3.45 mg, *p* < 0.001).

**Table 1:** Characteristics of the study population.

	Cancer group	Control group	*p*
Sample size	144	27,493	
Age (years)	64.67 ± 1.17	45.44 ± 0.24	<0.001
Caffeine intake (mg)	241.51 ± 18.96	183.08 ± 3.45	<0.001
Gender (male, %)	52.5	48.5	0.86
Race (%)			<0.001
Mexican American	1.4	8.6	
Non-Hispanic Black	9.5	11.6	
Non-Hispanic White	86.0	68.5	
Other Hispanic	1.7	4.8	
Other races	1.4	6.5	
Education (%)			0.29
Below high school	17.9	16.9	
High school	20.7	23.6	
Above high school	61.5	59.5	
PIR levels (%)			0.79
< 1.33	24.2	21.3	
1.33 ~ 3.5	30.7	32.2	
≥ 3.5	45.1	46.5	
Smoking (%)			<0.001
Current smoker	15.0	23.3	
Former smoker	49.2	22.8	
Never smoker	35.8	53.9	
Drinking (yes, %)	18.3	11.5	0.07

PIR, Poverty income ratio.

There was no significant difference in gender distribution (*p* = 0.86), with males representing 52.5% of the cancer group and 48.5% of the control group. The racial composition of the groups was significantly different (*p* < 0.001). Education levels (*p* = 0.29) and PIR levels showed no significant differences between the groups (*p* = 0.79). However, no significant difference was observed in alcohol consumption (*p* = 0.07), with 18.3% of the cancer group and 11.5% of the control group reporting alcohol consumption.

### 3.2 Weighted logistic regression analysis on the association between caffeine intake and colon cancer

Table 2 presents the weighted logistic regression analysis examining the association between caffeine intake and colon cancer. In Model 1, which was not adjusted for any covariates, the OR for colon cancer per 50 mg increase in caffeine intake was 1.04 (95% CI: 1.02–1.05, *p* < 0.001), indicating a significant positive association between caffeine intake and colon cancer risk.

**Table 2:** Weighted association between caffeine intake and colon cancer based on logistic regression.

	Model 1	*p*	Model 2	*P*	Model 3	*p*
	OR (95% CI)		OR (95% CI)		OR (95% CI)	
Caffeine (Per 50 mg)	1.04 (1.02, 1.05)	<0.001	1.04 (1.02, 1.06)	<0.001	1.04 (1.02, 1.06)	<0.001
Caffeine (Categories)						
Q1	Reference		Reference		Reference	
Q2	0.41 (0.21, 0.83)	0.013	0.46 (0.23, 0.92)	0.028	0.48 (0.24, 0.96)	0.039
Q3	0.71 (0.37, 1.37)	0.307	0.67 (0.35, 1.29)	0.224	0.67 (0.35, 1.30)	0.235
Q4	2.09 (1.21, 3.60)	0.009	1.97 (1.14, 3.42)	0.016	2.00 (1.11, 3.59)	0.022

Model 1 adjusted for no covariates. Model 2 adjusted for age, gender, and race. Model 3 adjusted for age, gender, race, education, poverty income ratio levels, smoking, drinking, and diabetes.

In Model 2, which was adjusted for age, gender, and race, the OR for colon cancer per 50 mg increase in caffeine intake remained significant at 1.04 (95% CI: 1.02–1.06, *p* < 0.001). The analysis of caffeine intake by categories revealed a significantly lower risk of colon cancer in the second quartile (Q2) compared with the first quartile (Q1) as the reference group (OR: 0.46, 95% CI: 0.23–0.92, *p* = 0.028). The risk of colon cancer in the third quartile (Q3) was not statistically significant compared to the reference group (OR: 0.67, 95% CI: 0.35–1.29, *p* = 0.224). However, the fourth quartile (Q4) showed a significantly higher risk of colon cancer compared to the reference group (OR: 1.97, 95% CI: 1.14–3.42, *p* = 0.016).

In Model 3, which was further adjusted for education, poverty income ratio levels, smoking, drinking, and diabetes, the OR for colon cancer per 50 mg increase in caffeine intake remained significant at 1.04 (95% CI: 1.02–1.06, *p* < 0.001). The risk of colon cancer in Q2 was significantly lower compared to the reference group (OR: 0.48, 95% CI: 0.24–0.96, *p* = 0.039), while the risk in Q3 was not statistically significant (OR: 0.67, 95% CI: 0.35–1.30, *p* = 0.235). The risk of colon cancer in Q4 was significantly higher compared to the reference group (OR: 2.00, 95% CI: 1.11–3.59, *p* = 0.022).

### 3.3 Dose–response association analysis

**Figure 1:**
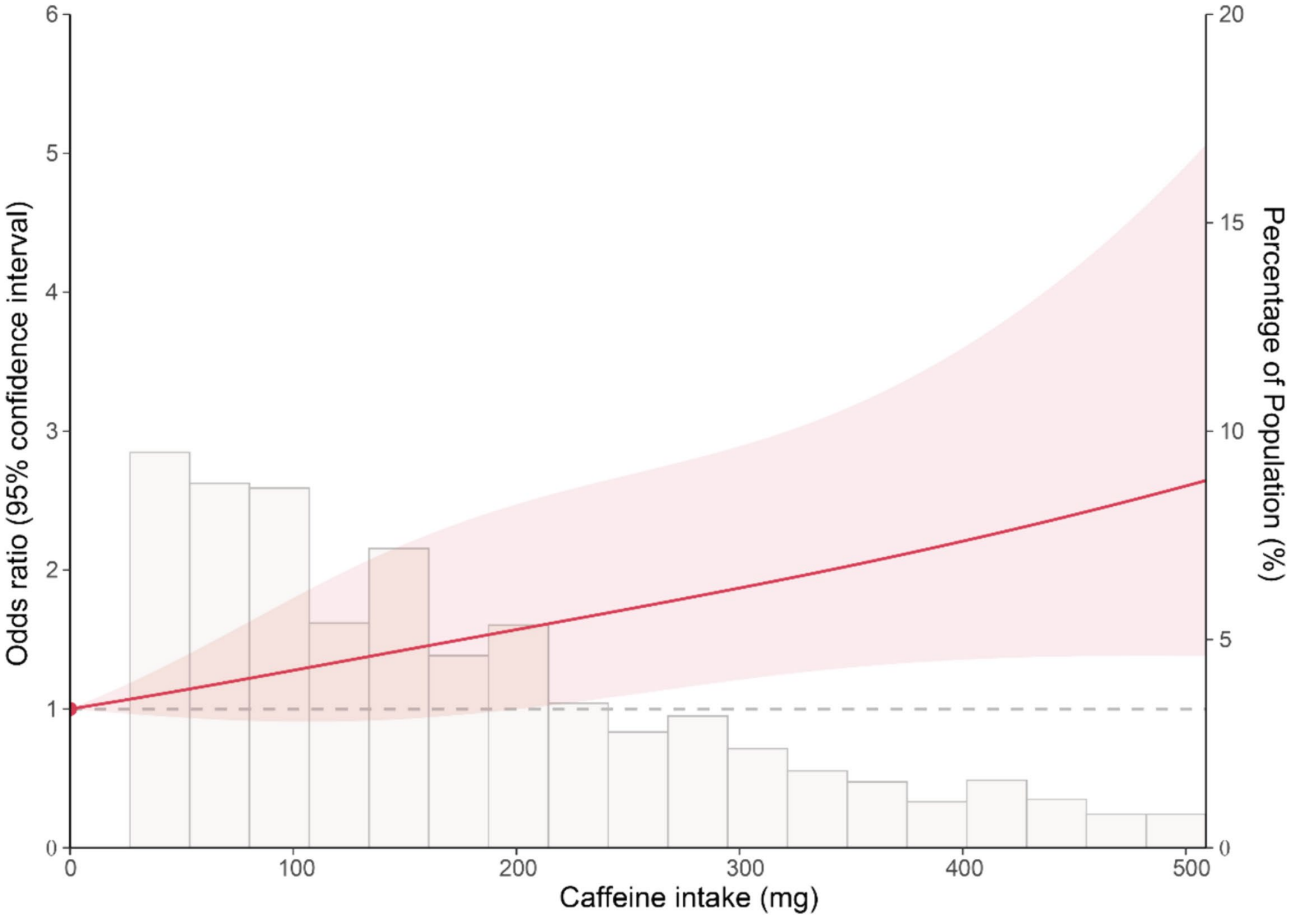
Restricted cubic spline plot of the association between caffeine intake and the risk of colon cancer. The solid line represents the OR of colon cancer for varying levels of caffeine intake, with 0 mg caffeine intake set as the reference. The shaded area represents the 95% confidence interval.

To explore the dose–response association between caffeine intake and the risk of colon cancer, we conducted the restricted cubic spline analysis based on logistic regression. The caffeine intake of 0 mg was set as the reference. As shown in Figure 1, the results reveal a non-significant association between caffeine intake and the risk of colon cancer at low levels. However, at higher levels of caffeine intake, a significant association was observed. The overall *p*-value for the association was 0.007, indicating a statistically significant relationship between caffeine intake and the risk of colon cancer. This indicates that the dose–response relationship between caffeine intake and the risk of colon cancer is predominantly driven by the association observed at higher levels of caffeine intake.

## 4 Discussion

This study explored the association between caffeine intake and the prevalence of colon cancer utilizing data from the NHANES survey from 2001 to 2014. Our findings indicated a significant association between higher caffeine intake and an increased risk of colon cancer. This association remained significant adjusting for age, gender, race, education, poverty income ratio levels, smoking, drinking, and diabetes. Additionally, we observed a reduced risk of colon cancer at low levels of caffeine intake, suggesting that the relationship may be influenced by varying consumption levels. Moreover, our dose–response analysis using restricted cubic spline regression revealed the trend in the relationship between caffeine intake and colon cancer risk, with a significant association observed at higher levels of caffeine intake. These findings enhanced the understanding of the relationship between caffeine intake and colon cancer risk and provided valuable insights that could guide public health recommendations and risk assessment strategies.

Research on the association between coffee consumption and colon cancer risk has yielded conflicting results. A meta-analysis encompassing 26 prospective studies with a collective total of 3,308,028 subjects, revealed a protective effect of coffee against colorectal cancer in the U.S. population, with a risk ratio of 0.83 (95% CI: 0.72–0.95). However, this analysis found no significant association between coffee intake and rectal cancer (16). Caroline et al. (21) investigated the association between coffee intake and the risk of colorectal cancer in older US adults, encompassing 47,010 men and 60,051 women aged 47–96 years without prior cancer diagnosis. Their findings indicated that consuming ≥2 cups of decaffeinated coffee a day was associated with a decreased risk of colorectal cancer (hazard ratio = 0.82, 95% CI = 0.69 ~ 0.96), colon cancer (hazard ratio = 0.82, 95% CI = 0.69 ~ 0.99), and rectal cancer (hazard ratio = 0.63, 95% CI: 0.40 ~ 0.99) compared to caffeinated coffee. Giovannucci’s meta-analysis, which combined data from 12 case–control studies and 5 cohort studies, demonstrated an inverse relationship, with a pooled relative risk of 0.72 (95% CI: 0.61–0.84) (22). Similarly, the NIH-AARP Diet and Health Study revealed a significant decrease in colon cancer risk among individuals consuming 4/5 cups (HR: 0.85; 95% CI: 0.75–0.96) and ≥ 6 cups (HR: 0.74; 95% CI: 0.61–0.89) of coffee daily (23). Based on 5,145 cases and 4,097 controls from the MECC study, Schmit et al. (24), found that coffee consumption reduced the risk of colorectal cancer by 26%. The HERPACC-I and II studies further supported these findings, showing that consuming three or more cups of coffee daily was associated with a reduced risk of colon cancer (OR: 0.78; 95% CI: 0.65–0.92) in Asian populations (25).

In contrast, a pooled analysis of 13 prospective cohort studies indicated that consumption of more than 1,400 g of coffee daily did not increase colon cancer risk (RR: 1.07; 95% CI: 0.89–1.30) (26). Furthermore, data from the Nurses’ Health Study and the Health Professionals’ Follow-up Study showed no significant association between caffeinated coffee consumption and the risk of colon or rectal cancer (pooled HR: 0.99; 95% CI: 0.96–1.03) (27). A meta-analysis of 12 additional cohort studies also found no significant impact of coffee consumption on colorectal cancer risk (28). These studies highlight that it is necessary to further investigate the associations between caffeinated and decaffeinated coffee intake and colorectal cancer risk.

Our study adds to the expanding body of knowledge regarding the association between caffeine intake and colon cancer risk. Our results suggest that higher levels of caffeine consumption may be linked to an increased risk of colon cancer, underscoring the potential need for public health initiatives to curtail excessive caffeine consumption. To further delineate the causal relationship between caffeine intake and colon cancer risk, future research should employ longitudinal study designs. Additionally, exploring the biological mechanisms behind this association and how it may be modified by factors such as genetics, sex, and lifestyle habits could offer valuable insights for developing effective cancer prevention strategies.

The strengths of our study include the use of a large, nationally representative dataset, which enhances the generalizability of our findings to the US adult population. Additionally, the comprehensive adjustment for potential confounding factors minimizes the risk of residual confounding. Moreover, the application of restricted cubic spline regression in our dose–response analysis allowed for a more flexible representation of the relationship between caffeine intake and colon cancer risk. However, our study also has some limitations. First, the cross-sectional nature of NHANES data precludes any determination of causality between caffeine intake and colon cancer risk. Second, the reliance on self-reported dietary information and cancer diagnoses may have introduced recall and reporting biases. Third, despite adjusting for multiple confounders, there is still a possibility of residual confounding (such as family history of colorectal cancer in first-degree relatives, history of abdominal radiation, and inflammatory bowel disease) due to unmeasured or inadequately measured factors. Lastly, the single 24-h dietary recall used to estimate caffeine intake may not accurately represent habitual consumption patterns.

## 5 Conclusion

In summary, our findings provide evidence for a positive association between high levels of caffeine intake and the risk of colon cancer among US adults. The dose–response relationship analysis, utilizing restricted cubic spline regression, revealed a predominantly linear relationship, with a significant association observed at higher levels of caffeine intake. These findings suggest that excessive caffeine consumption may be a risk factor for colon cancer. Future longitudinal studies are warranted to confirm our findings and to further investigate the potential mechanisms underlying the observed association. Public health efforts to promote moderate caffeine consumption and to raise awareness of the potential risks associated with excessive intake may help to reduce the incidence of colon cancer in the population.

## Data availability statement

Publicly available datasets were analyzed in this study. This data can be found at: https://www.cdc.gov/nchs/nhanes/index.htm.

## Ethics statement

The survey protocol, including study design and data collection procedures, is approved by the National Center for Health Statistics Ethics Review Board, and all the participants provided the written informed consent. The studies were conducted in accordance with the local legislation and institutional requirements. Written informed consent for participation was not required from the participants or the participants' legal guardians/next of kin in accordance with the national legislation and institutional requirements.

## Author contributions

YQ: Conceptualization, Formal analysis, Investigation, Methodology, Software, Visualization, Writing – original draft, Writing – review & editing. YC: Conceptualization, Formal analysis, Investigation, Methodology, Software, Validation, Visualization, Writing – original draft, Writing – review & editing. FC: Conceptualization, Formal analysis, Investigation, Methodology, Project administration, Supervision, Validation, Writing – original draft, Writing – review & editing.
